# Risk factors for musculoskeletal disorders among takeaway riders: Up-to-date evidence in Shanghai, China

**DOI:** 10.3389/fpubh.2022.988724

**Published:** 2022-11-21

**Authors:** Ziyun Li, Xiaochen Bo, Chen Qian, Mingyue Chen, Yuqing Shao, Yuxun Peng, Ruian Cai, Xiaojing Huang, Lijun Wei, Jinzhong Zhao, Jianwei Shi

**Affiliations:** ^1^Department of Sports Medicine, Shanghai Sixth People's Hospital Affiliated to Shanghai Jiao Tong University School of Medicine, Shanghai, China; ^2^School of Public Health, Shanghai Jiaotong University School of Medicine, Shanghai, China; ^3^School of Management, Xuzhou Medical University, Xuzhou, China; ^4^Shanghai Minhang Wujing Community Healthcare Center, Shanghai, China; ^5^Department of General Practice, Yangpu Hospital, Tongji University School of Medicine, Shanghai, China; ^6^Department of Social Medicine and Health Management, School of Public Health, Shanghai Jiaotong University School of Medicine, Shanghai, China

**Keywords:** work-related factors, musculoskeletal disorders (MSDs), influence, risk factor, takeaway

## Abstract

**Background:**

Musculoskeletal disorders (MSDs) are common occupational diseases. However, the influencing mechanisms were not clear in the new emerging takeaway rider occupation in the catering industry in China.

**Methods:**

A cross-sectional study was conducted using a takeaway rider sample from one of the largest e-platforms, the Mei Tuan Company in Shanghai. The chi-square test was used to compare the sex differences in MSDs according to various factors. Binary logistic regressions were then performed to explore the potential risk factors for the occurrence and severity of MSDs adjusted by age, sex and vehicle type. Crude odds ratios (CORs) and adjusted odds ratios (AORs) and their 95% confidence intervals (CIs) for predictors were reported.

**Results:**

The prevalence of MSDs was found to be 54.9% (*n* = 361). Shoulders (joint pain: 24.5%, *n* = 154/629 cases; muscle pain: 29.0%, *n* = 183/632 cases; muscle numbness: 31.7%, *n* = 120/379 cases) and neck (joint pain: 17.0%, *n* = 107/629 cases; muscle pain: 14.1%, *n* = 89/632 cases; muscle numbness: 15.3%, *n* = 58/379 cases) were the most affected regions. Irregular meals (often having regular meals: *p* = 0.03, AOR = 1.89, 95% CI: 1.05–3.39; sometimes: *p* < 0.01, AOR = 2.54, 95% CI: 1.49–4.34 and seldomly: *p* < 0.01, AOR = 4.24, 95% CI: 2.28–7.91) were positively associated with the occurrence of MSDs. Work-related factors, including working over 5 years (*p* = 0.02, AOR = 1.87, 95% CI: 1.10–3.17) and over 51 km of food delivery distance per day (51–75 km: *p* = 0.02, AOR = 2.13, 95CI%:1.13–4.01; ≥76 km: *p* < 0.01, AOR = 3.12, 95CI%: 1.44–6.77), were strongly associated with severity.

**Conclusion:**

MSDs were common among takeaway riders. Personal lifestyles (meal irregularity) were found to predict the occurrence, while work-related factors (longer years of employment and prolonged food delivery distance) were positively associated with severity. Public health efforts should be made to prevent MSDs in this population.

## Introduction

Musculoskeletal disorders (MSDs) are a series of common occupational-related disorders in which work exposures play a contributing causal role (1). MSDs can result from cumulative microdamage induced by risk factors at the cellular and/or tissue level over time and include inflammatory and degenerative diseases such as knee osteoarthritis, hip osteoarthritis, and frozen shoulder (2–4). The diseases lead to decreased productivity, work-time loss, or work leave and ultimately result in a large health resource burden (2, 3). Hence, there is a fast-expanding body of knowledge and attention focused on MSDs, as they present a serious threat to public health and place a financial burden on health insurance programs, businesses, and workers (5).

In recent years, takeaway rider occupation in the catering industry has become an emerging vocation and is rapidly increasing in popularity as a result of new business economies in China and other countries (6, 7). Catering service is realized mainly by customers ordering food online, offline merchants preparing food, and takeaway riders delivering food (8). Physical demands for takeaway riders include standing for a prolonged time, lifting items frequently, carrying the take-away food and transferring food from one place to another. As a result, takeaway riders may be susceptible to MSDs.

Sekkay et al. (9) investigated short- and long-distance industrial gas delivery truck drivers in Canada and discovered that among drivers reporting musculoskeletal pain in the past 12 months, the areas with the highest prevalence were the low back (21.1%), shoulders (20.3%), and neck (14.6%). For those reporting musculoskeletal pain in the past 7 days, the areas with the highest prevalence were also the low back (14.6%), shoulders (13.8%), and neck (8.9%) (9). The study found that high effort-reward imbalance, working with hands above shoulders, hand-arm vibration and whole-body vibration were risk factors for MSDs (9). However, the research subjects were truck drivers, whose vehicles are different from those of takeaway riders in the catering industry. Currently, studies of the MSDs of takeaway riders in the catering industry are relatively scarce. Yang et al. (8) investigated 137 takeaway riders in the catering industry with 49.6% (68/137) aged ≤25 years and found that the incidence of MSDs was 67.9%, with the highest prevalence in the neck (35.8%, 49/137) and shoulders (35.8%, 49/137), followed by the lower back (34.3%, 47/137), waist and thigh (34.3%, 47/137) and knee (28.5%, 39/137) (8). In Yang et al.'s (8) study, it was found that hand-arm vibrations were a risk factor for the occurrence of MSDs.

A wide range of work-related factors have already been acknowledged as important risk factors for MSDs, including vibration, prolonged working hours, and posture demands (2, 9–12). Furthermore, personal lifestyle could also greatly influence MSDs. An unhealthy personal lifestyle (including harmful use of tobacco/alcohol, unhealthy food habits and resistance to physical exercise) has been proposed as a risk factor for MSDs, while healthy personal lifestyles such as nap habits are negatively related to MSDs (1, 13).

In conclusion, although factors related to lifestyle and work were considered to be associated with MSDs among various occupational workers, no known research has studied the effects of these risk factors on MSDs for takeaway riders. Few previous papers have investigated the detailed distribution of MSDs symptoms among takeaway riders; also, the risk factors for MSDs occurrence and severity among takeaway riders are unclear. Therefore, this study innovatively investigated the MSDs of takeaway riders in the catering industry in China. The objective was to systematically describe the occurrence of MSDs, the influencing mechanism of possible factors on their occurrence and the severity among takeaway riders in the catering industry in Shanghai, China.

## Methods

### Data sources

In this study, the sample was recruited from the Mei Tuan Company in Shanghai, which is a service e-commerce platform, with a takeaway service as its primary service. By the end of 2019, the total number of takeaway riders reached 3.987 million for one of the largest businesses, the Mei Tuan company, which mainly operated in Beijing, Shanghai, Shenzhen and other large cities (8). Participant recruitment was conducted with the assistance of Mei Tuan's human resources personnel, and the participants involved were randomly chosen through the extraction of their job numbers. The inclusion criteria were as follows: (1) relevant work experience (over a month working) as takeaway riders and (2) voluntary participation in the survey. From July to August 2021, an online survey was conducted. All participants in this study placed food into a fixed container fixed on the baggage rack without shoulder straps. The questionnaire was named “Health investigation of takeaway riders in Shanghai,” with 27 multiple-choice questions included, and it was developed from standardized MSD questionnaires [including the Cornell Musculoskeletal Discomfort Questionnaire, the International Knee Documentation Committee Knee Evaluation Form and the Nordic-Musculoskeletal Questionnaire (14, 15)]. Considering the sample chosen principle, according to the statistical requirement, we first calculated a minimum size of 10 times the number of items in the questionnaire (16). As a result, the 27-item questionnaire required a minimum sample size of 270. Then, we decided to expand the sample to two times 270, which is 540. During our study, before the formal investigation, we conducted a pilot study in June 2021, which covered 100 questionnaires in the Yangpu district, Shanghai. Sixty-nine effective questionnaires were obtained in the pilot study, and the corresponding effective rate was 0.69. Then, for the formal investigation, the final number of questionnaires was calculated and set to 540/0.69 = 783. Trained investigators were assigned to explain all the questions at the questionnaire distributing sites. A total of 783 questionnaires were distributed, and 657 effective questionnaires were obtained (exclusion criteria included missing values >10%, answers that tended to be consistent, etc.), and the effective rate was 83.9%.

### Measures

#### Independent variables

In our study, demographic variables included sex, age, height, weight, location of registered household, education level, total monthly family income, type of medical insurance, marital status, the status of living with family and willingness to obtain medical service (17). Additionally, lifestyle variables included regular consumption of meals (always or often or sometimes or seldom or never) and daily sleeping duration (≤6 h or 7–8 h or ≥9 h), in which always, often, sometimes, seldom and never indicated every day, once every 2 to 3 days, once a week, once every 2 or 3 weeks and never. Additionally, the work-related variables were measured, including working status (part-time or full-time), years of employment (< 5 years or ≥5 years), daily working hours (< 8 or 8–10 h or 11–13 or ≥14 h), daily number of floors climbed (≤10 floors or 11–20 floors or 21–30 floors or ≥31 floors), vehicle type (bicycles or motorcycles/battery powered bikes or vans/cars or other) and daily food delivery distance (≤25 or 26–50 km or 51–75 or ≥76 km).

### Outcome variables

In this study, we measured the various statuses of musculoskeletal symptoms, including (1) the occurrence of symptoms (joint pain, muscle pain and muscle numbness), the injured regions and the duration (duration: never, < 1 month, 1–5 months, 6–11 months, 1–2 years and >3 years). Questions involving different symptoms were separately raised. As shoulder joint and muscle pain could be hard to extinguish, we identified joint pain as pain in a deeper site indicating injury in the rotor cuff, scapula, head of humerus and ligaments around the shoulder joint (potentially accompanied by a reduced range of motion), while muscle pain would be more superficial, indicating injuries in the deltoid muscle, latissimus dorsi, and trapezius muscle (18, 19). All information was provided by the participants. (2) Additionally, respondents with MSDs symptoms were selected, and the severity of musculoskeletal symptoms, showing MSDs' impacts on daily life, was measured by a standardized rating scale (Numerical Rating Scale). Scores above four indicated severe disorders, while scores between 1 and 3 indicated minor disorders (20).

### Adjusting variables

Adjusting variables were collected, including sex, age and vehicle type. Age was categorized into 4 groups (18–24 years, 25–30 years, 31–40 years and >40 years) based on WHO suggestions for Asian regions and the internal distribution (21).

### Statistical analysis

SPSS software (SPSS 25.0, Chicago, IL, USA) was used for data analysis. First, we described demographic factors, lifestyle factors, working conditions and musculoskeletal symptom information. Then, the chi-square test was performed to compare the differences in various factors between sexes. Finally, two binary logistic regression models were constructed to explore potential risk factors for occurrence and severity, and both were adjusted by age, sex and vehicle type. Crude odds ratios (CORs) and adjusted odds ratios (AORs) and their 95% confidence intervals (CIs) for predictors were reported.

## Results

### Participant demographics, lifestyle factors, and work-related factors

As shown in Table 1, of all 657 respondents, 70.9% (*n* = 466) were male, and most were aged 18–24 years (*n* = 317, 48.3%) and had a normal BMI (*n* = 373, 56.8%). Most were unmarried (*n* = 439, 66.8%), lived with their family members (*n* = 417, 63.5%), and had a household registration outside Shanghai (*n* = 459, 69.9%). A total of 140 respondents (21.3%) did not finish senior high school, and 152 (23.1%) had a monthly household income lower than 5,000 RMB. Almost all respondents were willing to seek medical service (*n* = 563, 85.7%), but 10.81% (*n* = 71) did not have any kind of medical insurance. Regarding personal lifestyles, only 13.4% (*n* = 88) always consumed meals regularly, and 54.3% (*n* = 357) had adequate sleep, with a 7–8 h sleeping duration. Regarding work-related factors, most of the participants had part-time jobs (*n* = 397, 60.4%) and were employed for <5 years (*n* = 493, 75.0%). The majority preferred motorcycles or battery-powered bikes (*n* = 539, 82.04%), and only 46.0% (*n* = 302) worked <8 h per day (Table 1).

**Table 1:** Distribution of demographics and possible influencing factors.

**Variables classification**	**Total (*N* = 657)**	**Male (*N* = 466)**	**Female (*N* = 191)**	***p*-values**
**Demographics**				
**Age**				
18–24 years	317 (48.3%)	238 (51.1%)	79 (41.4%)	<0.01
25–30 years	182 (27.7%)	127 (27.3%)	55 (28.8%)	
31–40 years	104 (15.8%)	76 (16.3%)	28 (14.7%)	
>40 years	54 (8.2%)	25 (5.4%)	29 (15.2%)	
**BMI**				
Underweight (< 18.5)	65 (9.9%)	28 (6.0%)	37 (19.4%)	<0.01
Normal (18.5–23.9)	373 (56.8%)	264 (56.7%)	109 (57.1%)	
Overweight (24–27.9)	153 (23.3%)	121 (26.0%)	32 (16.8%)	
Obese (≥28)	66 (10.1%)	53 (11.4%)	13 (6.8%)	
**Household registration**				
Shanghai	198 (30.1%)	142 (30.5%)	56 (29.3%)	0.77
Other cities	459 (69.9%)	324 (69.5%)	135 (70.7%)	
**Education**				
Junior high school or below	140 (21.3%)	100 (21.5%)	40 (20.9%)	0.2
Senior high school	154 (23.4%)	119 (22.5%)	35 (18.3%)	
Junior college	159 (24.2%)	110 (23.6%)	49 (25.7%)	
Bachelor's degree or above	204 (31.1%)	137 (29.4%)	67 (35.1%)	
**Monthly household income (RMB)**				
< 5,000	152 (23.1%)	92 (19.7%)	60 (31.4%)	0.01
5,000–9,999	249 (37.9%)	190 (40.8%)	59 (30.9%)	
≥10,000	187 (28.5%)	137 (29.4%)	50 (26.2%)	
Unknown	69 (10.5%)	47 (10.1%)	22 (11.5%)	
**Medical insurance**				
Shanghai medical insurance	197 (30.0%)	142 (30.5%)	55 (28.8%)	0.39
Other cities medical insurance	188 (28.6%)	125 (26.8%)	63 (33.0%)	
New rural cooperative medical insurance	92 (14.0%)	65 (13.9%)	27 (14.1%)	
Commercial medical insurance	109 (16.6%)	78 (16.7%)	31 (16.2%)	
No medical insurance	71 (10.8%)	56 (12.0%)	15 (7.9%)	
**Marital status**				
Unmarried	439 (66.8%)	334 (71.7%)	105 (55.0%)	<0.01
Married	165 (25.1%)	98 (21.0%)	67 (35.1%)	
Divorced/Widowed	53 (8.1%)	34 (7.3%)	19 (9.9%)	
**Living with family members**				
Yes	417 (63.5%)	287 (61.6%)	130 (68.1%)	0.12
No	240 (36.5%)	179 (38.4%)	61 (31.9%)	
**Willingness to seek medical service**				
Yes	563 (85.7%)	404 (86.7%)	159 (83.2%)	0.25
No	94 (14.3%)	62 (13.3%)	32 (16.8%)	
**Lifestyle variables**				
**Regularity of meals**				
Always	88 (13.4%)	66 (14.2%)	22 (11.5%)	<0.01
Often	128 (19.5%)	75 (16.1%)	53 (27.7%)	
Sometimes	227 (34.6%)	154 (33.0%)	73 (38.2%)	
Seldom	113 (17.2%)	87 (18.7%)	26 (13.6%)	
Never	101 (15.4%)	84 (18.0%)	17 (8.9%)	
**Daily sleep duration**				
≤6 h	218 (33.2%)	143 (30.7%)	75 (39.3%)	0.02
7–8 h	357 (54.3%)	256 (54.9%)	101 (52.9%)	
≥9 h	82 (12.5%)	67 (14.4%)	15 (7.9%)	
**Work-related variables**				
**Working status**				
Full-time	260 (39.6%)	200 (42.9%)	60 (31.4%)	0.01
Part-time	397 (60.4%)	266 (57.1%)	131 (68.6%)	
**Years of employment**				
<5 years	493 (75.0%)	345 (74.0%)	148 (77.5%)	0.35
≥5 years	164 (25.0%)	121 (26.0%)	43 (22.5%)	
**Daily working hours**				
<8 h	302 (46.0%)	197 (42.3%)	105 (55.0%)	0.01
8–10 h	263 (40.0%)	193 (41.4%)	70 (36.6%)	
11 13 h	69 (10.5%)	55 (11.8%)	14 (7.3%)	
≥14 h	23 (3.5%)	21 (4.5%)	2 (1.0%)	
**Daily number of floors climbed**				
≤10 floors	260 (39.6%)	169 (36.3%)	91 (47.6%)	<0.01
11–20 floors	193 (29.4%)	131 (28.1%)	62 (32.5%)	
21–30 floors	103 (15.7%)	78 (16.7%)	25 (13.1%)	
≥31 floors	101 (15.4%)	88 (18.9%)	13 (6.8%)	
**Vehicle type**				
Bicycles	72 (11.0%)	59 (12.7%)	13 (6.8%)	0.02
Motorcycles/battery powered bikes	539 (82.0%)	373 (80.0%)	166 (86.9%)	
Vans/cars	37 (5.6%)	30 (6.4%)	7 (3.6%)	
Other	9 (1.4%)	5 (2.6%)	4 (0.9%)	
**Daily food delivery distance**				
≤25 km	214 (32.6%)	135 (29.0%)	79 (41.4%)	<0.01
26–50 km	168 (25.6%)	119 (25.5%)	49 (25.7%)	
51–75 km	154 (23.4%)	113 (24.2%)	41 (21.5%)	
≥76 km	121 (18.4%)	99 (21.2%)	22 (11.5%)	

Compared to males, age (*p* < 0.01), BMI index (*p* < 0.01), monthly household income (*p* = 0.01), and marital status (*p* < 0.01) in female riders were significantly different from those in males. Regarding lifestyle variables, regularity in meals (*p* < 0.01) and daily sleep duration (*p* = 0.02) were also different between the sexes. For work-related factors, female riders were more likely to have a part-time job (68.6 vs. 58.1%, *p* = 0.01), and differences were also found in daily working hours (*p* = 0.01), daily number of floors climbed (*p* < 0.01), vehicle type (*p* = 0.02) and daily food delivery distance (*p* < 0.01).

### Status of participants' MSDs

**Figure 1:**
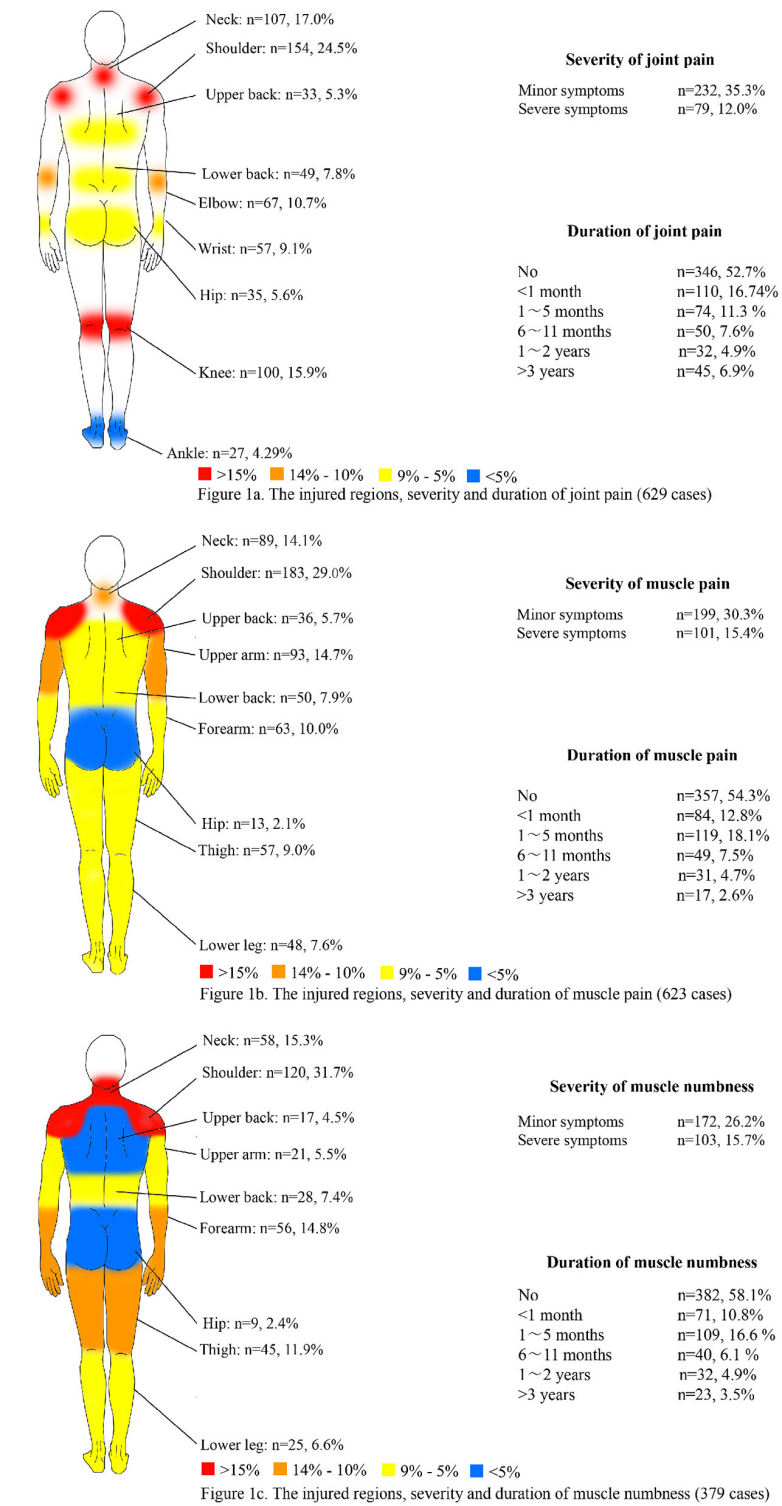
The injured regions, severity and duration of MSD symptoms.

The prevalence of MSDs in this study was 54.94% (*n* = 361). Each symptom was demonstrated as an injured region, and the severity and duration are shown in Figure 1. The top three regions with the highest prevalence of joint pain were the shoulder (24.5%, *n* = 154/629 cases), neck (17.0%, *n* = 107/629 cases) and knee (15.9%, *n* = 100/629 cases). For muscle pain, the top three regions were the shoulder (29.0%, *n* = 183/632 cases), upper arm (14.7%, *n* = 93/632 cases) and neck (14.1%, *n* = 89/632 cases). For muscle numbness, the top three regions were the shoulder (31.7%, *n* = 120/379 cases), neck (15.3%, *n* = 58/379 cases), and forearm (14.8%, *n* = 56/379 cases). Data of severity and duration are shown in Figure 1.

### Association between MSDs and possible influencing factors

Regarding the occurrence of symptoms, several influencing factors were associated with the occurrence of symptoms. Specifically, we found that those who often (*p* = 0.03, AOR = 1.89, 95% CI: 1.05–3.39), sometimes (*p* < 0.01, AOR = 2.54, 95% CI: 1.49~4.34) or seldomly (*p* < 0.01, AOR = 4.24, 95% CI: 2.28–7.91) ate regularly were more likely to be symptomatic than their counterparts (Table 2).

**Table 2:** Possible influencing factors of the occurrence of MSDs symptoms.

**Factors**	**Occurrence of symptoms**			
	**COR (95% CI)**	***p*-value**	AOR^*^(95% CI)	***p*-value**
**Lifestyle variables**				
**Regularity of meals**				
Always	ref.		ref.	
Often	1.83 (1.04–3.23)	0.04	1.89 (1.05–3.39)	0.03
Sometimes	2.52 (1.50–4.25)	<0.01	2.54 (1.49–4.34)	<0.01
Seldom	4.28 (2.32–7.89)	<0.01	4.24 (2.28–7.91)	<0.01
Never	1.19 (0.65–2.16)	0.58	1.15 (0.62–2.13)	0.66
**Daily sleep duration**				
≤6 h	1.24 (0.86–1.80)	0.25	1.25 (0.86–1.83)	0.24
7–8 h	ref.		ref.	
≥9 h	1.72 (1.01–2.91)	0.05	1.63 (0.94–2.80)	0.08
**Work-related variables**				
**Working status**				
Full-time	ref.		ref.	
Part-time	1.16 (0.81–1.66)	0.42	1.17 (0.81–1.69)	0.41
**Years of employment**				
<5 years	ref.		ref.	
≥5 years	1.14 (0.78–1.67)	0.50	1.07 (0.72–1.58)	0.73
**Daily working hours**				
<8 h	ref.		ref.	
8–10 h	1.36 (0.93–2.01)	0.12	1.34 (0.91–1.99)	0.14
11 13 hours	1.25 (0.69–2.27)	0.46	1.22 (0.67–2.24)	0.52
≥14 hours	1.12 (0.44–2.83)	0.81	1.14 (0.45–2.90)	0.78
**Daily number of floors climbed**				
≤10 floors	ref.		ref.	
11–20 floors	1.19 (0.78–1.81)	0.41	1.25 (0.81–1.92)	0.31
21–30 floors	1.19 (0.70–2.03)	0.52	1.21 (0.70–2.08)	0.50
≥31 floors	1.26 (0.71–2.22)	0.43	1.29 (0.72–2.31)	0.39
**Daily food delivery distance**				
≤25 km	ref.		ref.	
26–50 km	0.68 (0.43–1.10)	0.12	0.70 (0.43–1.14)	0.25
51–75 km	0.67 (0.43–1.05)	0.08	0.68 (0.43–1.07)	0.10
≥76 km	0.63 (0.37–1.08)	0.09	0.63 (0.36–1.08)	0.09

Note: ref.: reference;

* : adjusted for sex, age and vehicle type.

Regarding the severity of symptoms, the logistic regression showed that those who worked ≥5 years were 1.87 times more likely to have more severe symptoms than to have minor symptoms (*p* = 0.02, AOR = 1.87, 95% CI: 1.10–3.17). Additionally, long food delivery distance was a factor related to developing more severe symptoms, especially for the 51–75 km group (*p* = 0.02, AOR = 2.13, 95CI%:1.13–4.01) and the ≥76 km group (*p* < 0.01, AOR = 3.12, 95CI%: 1.44–6.77) (Table 3).

**Table 3:** Possible influencing factors of the severity of MSDs symptoms.

**Factors**	**Severity of symptoms**			
	**COR (95% CI)**	***p*-value**	AOR^*^(95% CI)	***p*-value**
**Lifestyle variables**				
**Regularity of meals**				
Always	ref.		ref.	
Often	1.64 (0.65–4.14)	0.30	1.87 (0.71–4.94)	0.21
Sometimes	0.91 (0.39–2.14)	0.82	1.06 (0.43–2.58)	0.91
Seldom	0.93 (0.38–2.31)	0.88	1.06 (0.41–2.73)	0.91
Never	1.75 (0.63–4.86)	0.29	1.66 (0.57–4.79)	0.35
**Daily sleep duration**				
≤6 h	1.14 (0.69–1.89)	0.62	1.08 (0.63–1.83)	0.78
7–8 h	ref.		ref.	
≥9 h	1.91 (0.98–3.73)	0.06	1.63 (0.80–3.30)	0.18
**Work-related variables**				
**Working status**				
Full-time	ref.		ref.	
Part-time	1.53 (0.92–2.54)	0.11	1.43 (0.84–2.44)	0.19
**Years of employment**				
<5 years	ref.		ref.	
≥5 years	2.00 (1.20–3.33)	0.01	1.87 (1.10–3.17)	0.02
**Daily working hours**				
<8 h	ref.		ref.	
8–10 h	0.76 (0.45–1.30)	0.32	0.72 (0.42–1.25)	0.25
11–13 h	1.30 (0.58–2.94)	0.52	1.45 (0.63–3.37)	0.39
≥14 h	2.47 (0.54–11.20)	0.24	2.60 (0.58–11.72)	0.21
**Daily number of floors climbed**				
≤10 floors	ref.		ref.	
11–20 floors	1.30 (0.73–2.30)	0.37	1.28 (0.70–2.34)	0.42
21–30 floors	1.49 (0.72–3.07)	0.28	1.48 (0.69–3.18)	0.31
≥31 floors	1.14 (0.52–2.51)	0.74	1.13 (0.50–2.55)	0.77
**Daily food delivery distance**				
≤25 km	ref.		ref.	
26–50 km	1.40 (0.74–2.65)	0.30	1.21 (0.61–2.38)	0.58
51–75 km	2.24 (1.22–4.12)	0.01	2.13 (1.13–4.01)	0.02
≥76 km	2.82 (1.35–5.92)	0.01	3.12 (1.44–6.77)	<0.01

Ref., reference;

* : adjusted for sex, age and vehicle type.

## Discussion

This research innovatively focused on the globally booming industry of delivery service and the prevention of MSDs among takeaway riders. We identified joint pain, muscle pain and muscle numbness as major symptoms and found that the prevalence was 54.95%, similar to that in Yang et al. (8) study on 150 takeaway riders (67.9%). The results showed that MSDs are a significant public health concern and are particularly prevalent among takeout riders. The prevalence in our research was slightly higher than that in Sekkay's et al. (9) study (43.1%), mainly because their research was focused on truck drivers, while the subjects in our study were mostly motorcycle or battery-powered bike riders (82.04%). Similarly, Matysiak et al. (22) found a prevalence of 57.37% in 337 police riders.

In our study, several hazards were identified for the takeaway riders. It was found that males and those below the age of 30 years old suffered more from MSDs. Most takeaway riders led a less healthy lifestyle, with 13.39% having meals regularly on a daily basis and only 54.34% having adequate sleeping habits. Regarding work-related information, high job mobility was observed in this population, with the majority of participants having part-time jobs (60.43%) and <5 years of employment (77.47%). Additionally, most of the participants preferred riding motorcycles or battery-powered bikes (82.04%). Regarding the MSDs, the most commonly injured regions in our study were the shoulders and neck, similar to the findings of Yang's study (8). However, Sekkay et al. found that the low back was the region with the highest prevalence, which might also be explained by the different types of vehicles used (9).

Regarding the occurrence of symptoms, unlike previous studies on MSDs that found work-related factors as risk factors, we discovered that intermittent irregular eating was an obvious risk factor. This could be explained by the younger age of takeaway riders. Contrary to other reports whose subjects were mostly middle-aged adults (1, 9), our respondents were mostly young adults aged 18–24 years who naturally are not in the peak age of degenerative diseases caused by cumulative micro damage (4, 23). Thus, riders' personal characteristics, namely, their long-term personal lifestyle, play a more important role in the onset of MSDs (24). A South Korean survey also found that temporary workers were more likely to skip meals than permanent workers, especially in the case of lunch (25). It is well-known that irregular meals do great harm to health, since skipping meals was associated with lower dietary quality as well as reduced healthy food purchases (26, 27) and thus led to obesity, diabetes, cardiovascular diseases and mental health problems (28, 29). Interestingly, when irregularity is then subdivided, intermittent irregular eating habits may cause more physical and psychological disorders by influencing metabolic and anxiolytic properties with consequent effects on daily activity levels due to the unreliable prediction of food availability (30). A study on surgeons also confirmed this finding that intermittent eating irregularly can lead to hypoglycaemia, electrolyte imbalance, psychological stress, sleep deprivation, and fatigue (31), which are all precursors of musculoskeletal injuries. Future studies will need to confirm these first findings in workers. Therefore, it can be suggested that personal lifestyle habits, especially a healthy diet, greatly affected the health of takeaway riders, and public health interventions should more actively encourage the adoption of mandatory breaks to improve the regularity of meal consumption to help reduce the risk of musculoskeletal disorders.

Our study adopted a rating scale to measure the severity of symptoms (20), and we discovered that although work-related factors are not associated with the onset of MSDs, they have a profound influence on the further deterioration of MSDs. Having been employed over 5 years and over 51 km of food delivery distance per day were found to be risk factors for severe symptoms. The harm associated with the workload may be related to the process of food delivery. Posture fatigue problems exist in motorcycle or battery-powered bike riders since a high volume of non-neutral postures in riding (e.g., excessive elongation of the neck) could lead to kinematic alterations in the shoulder, neck, lower body and spinal structures, causing muscle stiffness and pain and finally resulting in more severe MSDs (32, 33). Moreover, hand-arm vibration during the riding process made riders more inclined to grip handles, leading to increased static muscle activity in the arms, neck and shoulders, which was shown to harm the upper limbs in a meta-analysis (34). The vertical vibration caused by sitting on a backless seat could also injure the lower part of the body, since the hips and lower back absorb the vibration energy directly (35). Overall, prolonged years of employment and food delivery distance were associated with the severity of symptoms. On the basis of these results, health policies should focus more on lowering working hours and increasing working wages to reduce working load, as well as promoting ergonomic protection devices, thus protecting musculoskeletal systems.

There were some limitations in this study. First, the sample was from Shanghai, and a representative study needs to be based on a larger population from other regions in China. Second, there may be recall bias in the study, since symptomatic subjects might tend to recall more risk factors than their non-symptomatic counterparts.

## Conclusions

This research provides an innovative detailed examination of the MSDs of takeaway riders, which is an occupation that is rapidly developing in most countries. We discovered that the prevalence of MSDs was rather high among takeaway riders, and the most injured regions were the shoulders and neck. Additionally, personal lifestyles (meal irregularity) were found to be positively involved in the occurrence of musculoskeletal symptoms, and work-related factors (longer years of employment and prolonged food delivery distance) were more likely to predict an increase in symptom severity. Public health policies should pay more attention to increasing rest time and decreasing working hours among takeaway riders to establish better lifestyles and reduce working loads to help prevent MSDs.

## Data availability statement

The original contributions presented in the study are included in the article/supplementary material, further inquiries can be directed to the corresponding authors.

## Ethics statement

The studies involving human participants were reviewed and approved by the Ethics Committees of Tongji University (ref: LL-2016-ZRKX-017). The patients/participants provided their written informed consent to participate in this study.

## Author contributions

ZL, XB, and CQ performed the statistical analysis and drafted the manuscript. ZL, XH, LW, JZ, and JS participated in the design of the study and revision of the paper. ZL, XB, CQ, YS, YP, RC, and MC participated in the data collection. All authors contributed to the article and approved the submitted version.

## Funding

This study was supported by Shanghai Education Science Research Project (C2021039), Natural Science Foundation of China (71774116 and 71603182), Shanghai Public Health Outstanding Young Personnel Training Program (GWV-10.2-XD07), Soft Science Project of Shanghai Science and Technology Commission (22692107200), National Key Research and Development Program of China (2022YFC3601505), Shanghai Pujiang Program (2020PJC080), and College Student Innovation Training Program Project of Shanghai Jiao Tong University School of Medicine (1521Y043). The funding sources played no role in the design of this study or any role during its execution, analyses, data interpretation, or decision to submit results.

## Conflict of interest

The authors declare that the research was conducted in the absence of any commercial or financial relationships that could be construed as a potential conflict of interest.

## Publisher's note

All claims expressed in this article are solely those of the authors and do not necessarily represent those of their affiliated organizations, or those of the publisher, the editors and the reviewers. Any product that may be evaluated in this article, or claim that may be made by its manufacturer, is not guaranteed or endorsed by the publisher.

## Abbreviations

MSDs: musculoskeletal disorders.
